# Factors associated with underweight, overweight, stunting and wasting among primary school-going children participating in a school health initiative in South Africa

**DOI:** 10.1186/s40795-023-00778-x

**Published:** 2023-10-25

**Authors:** Netsai Bianca Gwelo, Joshua Sumankuuro, Olagoke Akintola, William R. Brieger

**Affiliations:** 1 School of Public Health, Faculty of Community and Health Sciences, University of the Western Cape, Cape Town, South Africa; 2 Department of Public Policy and Governance, Faculty of Public Policy and Governance, Dombo University of Business and Integrated Development Studies, Wa, SD Ghana; 3 School of Allied Health, Exercise and Sports Sciences, Faculty of Science and Health, Charles Sturt University, Orange, NSW Australia; 4 Bloomberg School of Public Health, Johns Hopkins University, Baltimore, MD USA

**Keywords:** Child nutrition, Integrated School Health Programme, Obesity, Stunting, Malnutrition, KwaZulu- Natal Province

## Abstract

**Background:**

The double burden of malnutrition among children remains a public health challenge in South Africa. In response, the government of South Africa developed the National Health Policy and Implementation Guidelines for school-going children in 2003. This policy was subsequently upgraded to ‘The Integrated School Health Programme’ in 2012. An element of the programme is the provision of a meal to school-going children on school days. However, evidence suggests that one-third of school-going children continue to have nutritional deficiencies. This study investigated the sociodemographic as well as the nutritional characteristics of school-going children participating in a school health initiative in KwaZulu-Natal Province, South Africa.

**Methods:**

This was a retrospective descriptive cross-sectional study involving 1,275 children (50.3% females and 49.7% males) aged 3 to 15 years. Epidemiological data on the screening of the children’s nutritional characteristics by school health nurses and school health nutritionists under the integrated school health programme (ISHP) was reviewed and analysed for the study.

**Results:**

Nearly half (50.3%) and 49.7% of the population were females and males, respectively. The average age of participants was 8.4 years old. ‘Underweight’ (p = 0.000), ‘overweight’ (p = 0.000), ‘at risk of overweight’ (p = 0.000),‘stunting’ (p = 0.000),‘severe stunting’ (p = 0.005), ‘wasting’ (p = 0.010), and ‘obesity’ (p = 0.037) were associated with the ‘schools that children attended’. School-going children’ living conditions were significantly associated with ‘normal weight’ (p = 0.000), ‘underweight’ (p = 0.000), and ‘underweight’ (p = 0.028). However, the social grant to parents/guardians had some positive effects on the percentage of children who reported ‘normal weight’ (55.4%), ‘wasting’ (1.0%), ‘underweight’ (4.0%), and ‘at risk of overweight’ (20.2%).

**Conclusion:**

Chronic nutritional deficiencies persist among children. Therefore, ISHP implementation must retarget specific regions of the country to ensure that national goals and gains on school-going children nutrition, are met and maintained. Indeed, considering the positive impact of the government’s social grant programme on the nutritional status of the children in this study, we recommend policy reforms that will increase parents’ and carers’ access to means of subsistence in order to meet the health and nutritional needs of children in the study communities.

**Supplementary Information:**

The online version contains supplementary material available at 10.1186/s40795-023-00778-x.

## Introduction

Globally, schools are seen as optimal settings for health promotion and interventions for children, adolescents, and the broader school community through school health programmes [1–3]. School health programmes provide an opportunity for health education and interventions aimed at removing health-related obstacles to learning. By fostering environments conducive to promoting health, school health interventions are a cost-effective method for enhancing children’s health and academic performance [2].

According to the annual South African Child Gauge, approximately 60% of South African children live below the poverty line, and about 6.4 million children live below the food poverty threshold [4, 5]. This report indicates that a significant number of children do not have access to the minimum amount of nutrition required. In South Africa’s educational policy, school health promotion has received a lot of attention. The priority was demonstrated through the review and improvement of the previous National Health Policy and Implementation Guidelines of 2003 to an Integrated School Health Programme (ISHP) [4]. Developed and implemented by the South African Department of Health in collaboration with the Department of Basic Education, the Department of Social Development, and non-governmental organisations, the Integrated School Health Programme (ISHP) aims to strengthen and expand existing school health promotion and school health services [6]. The programme emphasizes the holistic wellness of the child within the educational context [6]. According to policymakers, nutrition is a critical component of human existence, health, and development at all stages of the life course [6, 7]. The development of a child’s brain and nervous system, as well as their growth, organ formation and function, immune system, and brain and nervous system, are all dependent on adequate nutrition when they are young [8]. Typically, children between the ages of 3 and 18 are considered to be of school age. This is the optimal time to build up nutrient stores in preparation for the accelerated growth of adolescence, making the school-age years nutritionally significant [9]. Different age groups of school-going children constitute a vulnerable population that could be examined to understand the nutrition and health outcomes of school-going children in South Africa. ISHP is a comprehensive health package that aims to enhance the health and wellbeing of school children [10, 11]. The intervention in schools is especially opportune given the poor performance of child nutrition status indicators. For instance, according to the 2012 South African National Health and Nutrition Examination Survey (SANHANES), approximately 27% of children aged 1–9 in South Africa were stunted (i.e., had low height-for-age due to chronic malnutrition); 13% were overweight, and 5% were obese. Moreover, according to the 2016 South African Demographic and Health Survey (SADHS), 27% of children under the age of five were stunted [11, 12].

These nutritional status of school-aged children are a concern in both urban and rural communities of the nation, with dietary changes and sedentary lifestyles contributing to the problem. In South Africa, stunting, a symptom of chronic malnutrition, continues to be a significant problem among school-aged children [12]. According to the SANHANES 2012 survey, the prevalence of stunting among children aged 1 to 9 in KwaZulu-Natal province was approximately 26%, indicating a substantial proportion of children with low height-for-age as a result of chronic malnutrition [12]. Regarding the health concerns of school-going children, nutrition is a central component of the ISHP, with the objective of promoting healthy eating practices and facilitating access to nutritious foods [12, 13]. The objective of the school nutrition programme is to help address the nutritional needs of school-going children, especially, those from disadvantaged households.

During school sessions, the National School Nutrition Programme (NSNP) provides one nourishing meal daily to school-going children. The nutrition component is implemented by the NSNP, in collaboration with relevant governmental and non-governmental organisations. The policy aims to help develop multisectoral interlinked interventions that will improve nutritional outcomes and in synchronisation with the Sustainable Development Goal two (SDG 2) [14]. To provide innovative and community-based initiatives to aid in resolving social and economic concerns, such as poverty and malnutrition, experienced by citizens [15]. However, we did not find any study investigating the nutritional status of school-going children in schools receiving support from a non-governmental organization’s school health programme. This study examined the socio-demographic characteristics of school-going children and their nutritional status in the integrated school health programme in the Marianhill area of Durban, South Africa.

## Methods

### Research setting and context

The study was conducted in four primary schools participating in an integrated school health initiative (ISHI) in the Marianhill area located on the outskirts of Durban in KwaZulu-Natal province of South Africa. We have used pseudonyms: ‘School A’, “School B”, “School C’, and ‘School D’ to represent the four schools. These schools are located in a catchment area that is characterized by widespread poverty, high HIV incidence, and poor access to basic services and facilities [15, 16]. Further, the schools receive government subsidies for school fees because of the high levels of poverty in the communities [17]. A community-based organization has been providing health and social services in the Marianhill communities. This community-based care organization has also collaborated with different stakeholders in the implementation of an integrated school health programme in some schools. The community-based organization is the main implementer of the integrated school health programme in these four schools and was responsible for hiring and managing supplementary school health nurses and school health nutritionists to assess the health of children [13].

Further, school-going children were provided meals during lunch breaks from Monday to Friday on school days as part of the national school nutrition programme. The meals children receive at schools contribute to better nourishment and health for these children. The community-based organisation (CBO) also assists some orphans and vulnerable school-going children within the communities where they provide social services. These children were also participate in arogramme called ‘kitchen soup’. The programme is implemented by a community-based organisation, and it is independent of the integrated school health programme. In this programme, orphans and vulnerable children visit the CBO drop-in centres and they receive a meal after school. However, the drop-in centres only operate on weekdays.

### Research design

A retrospective cross-sectional descriptive design was adopted to examine the relationship between school-going children’s sociodemographic characteristics and their nutritional characteristics [18].

### Participants and sampling

A total of 1,275 children (50.3% girls and 49.7% boys) within the age range of 3 to 15 years were assessed and screened for nutritional health conditions by school health nurses. All children from all classes who consented to participating in the initiative were screened [19]. See Babatunde and Akintola [19], for a detailed description of eligibility and screening processes.

### Data collection

We collected epidemiological data by conducting retrospective chart reviews of school health screening reports and other documents. The cohort of school-going children in this study were the first batch of screening conducted by school health teams for 2017 (complete year). The school health teams comprised nurses, nutritionists, social workers, and school counselors among others, who receive appropriate training to provide healthcare. With regards to the school health nurses and school health nutritionists, WHO standard guideline for child growth as well as how to conduct anthropometric measurement of children’s nutritional characteristics forms part of their training. Meanwhile, the Department of Health also builds their capacity with regular courses and training workshops. The school health nurses provide essential healthcare to school children including regular screening. Specifically, all school-going children in the four primary schools were screened for different health conditions including: nutritional assessment, gross motor function; fine motor function; eye condition, oral health condition, ear condition, speech function, tuberculosis screening, deworming, immunisation, minor ailments, psychosocial problems and long-term health conditions. Thus, the school health team conducted a comprehensive assessment for each child on a weekly basis, completed an assessment form and also kept notes, records, and documents pertaining to the screening. The school health team comprises nurses, nutritionists, social workers, school counselors and other professionals, who may be co-opted to assist in providing some critical care [see Supplemental file used by school health nurses].

### Statistical rigour

To ensure accuracy, an Excel template was used to extract the data [20]. All recorded data on the nutritional assessment forms were extracted into the Excel template., Information included demographic characteristics of the school-going children, i.e., their age, gender, grade, school, parents’ economic status, and the children’s living arrangements. We also extracted data on the children’s nutritional health status i.e. underweight, obese, at risk of overweight, stunting, wasting, severe stunting, and underweight. These measures were predetermined in the data set obtained for the study. The data extraction was conducted on all records of these children and health screening records on the children were included in the analysis. This was done to prevent selective bias and ensure a comprehensive and thorough examination of children’s nutritional status, relative to their sociodemographic characteristics. Data was double entered in Microsoft Excel sheet and cleaned for errors and missing values. The final dataset was thereafter imported into SPSS (v. 23) for the analysis.

### Anthropometric measurements

Anthropometric measurements are noninvasive quantitative measurements used in health promotion to assess nutritional status and general health in children. It is commonly used in pediatric populations to evaluate growth, developmental patterns, and nutritional adequacy. Core elements of anthropometry include height, weight, head circumference, BMI, body circumferences (such as waist, hip, and limbs) to assess adiposity, and skinfold thickness [4, 21]. Thus, in the study schools, the school health teams received refresher training from nutritionists to prepare for the successful implementation of the school health nutrition promotion initiative. The training covered ethics, anthropometric measurements, and screening data collection, in accordance with WHO standards guidelines [22]. The children’ s weight and height were measured and body mass index-for-age and height-for-age z-scores were computed according to World Health Organization growth standards in order to determine the prevalence of underweight, overweight, obesity and stunting. Waist circumference was measured to classify the children as having a high or very high risk for metabolic disease. The school health team of trained nurses conducted the measurement and data collection. From the school health team, the children were weighed in kilograms (kgs) without shoes and jerseys using a digital portable scale. Weight was measured to the nearest 0.1 kg. Height was measured in centimeters (cm) to the last completed 0.1 cm using a portable stadiometer. The same measurement protocols were used for all children. Body mass index (BMI) was calculated as weight per square meter of height (kg/m^2^). The diagnosis of a child’s nutritional status (underweight, stunting, wasting, severe stunting), was based on the WHO standard reference chart booklet for: height for age, weight for age, BMI for age (see WHO Multicentre Growth Reference Study Group, 2006 [22]). The BMI for age and sex of each child was compared with the SD “standard deviation” percentiles (z-scores) of weight for age <-2SD, weight-for-height > + 2 SD, height for age <-2SD, and BMI for age. According to the WHO Child Growth Standards, these indicators are anthropometric z scores (WHO Multicentre Growth Reference Study Group, 2006) [22]. When the data was extracted, we followed Adedokun and Yaya’s [23] article in conducting the analysis.

### Data processing and analysis

We reviewed and extracted data from all the children’s assessment forms and reports documented during the assessment process. The data already entered into the Microsoft Excel template was later imported to the Statistical Package for the Social Scientists (SPSS v. 23) for analysis. Continuous variables were summarized using mean and standard deviation. Categorical variables were presented in frequencies and percentages. Pearson’s chi-square (χ^2^) test was used to assess the level of independence of the variables. Fisher’s exact test was reported for cell count of less than 5. Statistical analysis was conducted at 95% confidence interval and a *p*-value of less than 0.05 was considered statistically significant. We had intended to do further analysis using logistic regression modelling. However, we found that the category of interest [‘Yes’] for the outcome variables had small frequencies to support the analysis. In such statistical dilemma, Scott Long and Jeremy freeze [24] indicate that the maximum likelihood estimation including logistic regression with less than 100 cases is “risky,”and that 500 cases is generally “adequate”. Long further note that there should be at least 10 cases per predictor. Therefore, we followed Ivan Elisabeth Purba, Agnes Purba, Rinawati Sembiring [25] and Walsh et al. [26] in conducting and presenting the data analysis. Of the 21 variables in Walsh et al’s [26] study, logistic regression model was constructed for only three (3) variables that showed adequate frequencies and statistical significance (at chi-square test of independence).

### Findings

We present the results on sociodemographic characteristics of school-going children and their nutritional status, using charts, frequencies, and percentages. Chi-square test of independence was also conducted to examine the level of significance of the association among the sociodemographic characteristics and nutritional status.

### Sociodemographic characteristics of the children

We present the sociodemographic characteristics of 1275 school-going children. The percentage of girls to boys was 50.3% and 49.7%, respectively. Mean age of participants was 8.44 (SD = 4.94) for both boys and girls. The majority of participants were within the ages of 6 to 9 years (n = 737, 57.8%) while only 9.8% (n = 125) of the participants were age 13 years and above [Table 1]. Most children were from ‘School D’ (n = 432, 33.9%) with 14.7% (n = 188) from ’School C’. The majority of the children were in Grade R (n = 256, 20.1%). School-going children whose parents/caregivers received social grants comprised 56.5%, and 28.1% lived with both parents, 19.2% lived with other relatives, 15.2% live with one parent whilst the majority (n = 590, 46.3%) did not specify their living arrangements [Table 1]. The results on parents/caregivers’ livelihood activities showed that 56.5% (n = 722) indicated that their parents/caregivers depended on other sources of livelihood and social grants. Those whose parents were both employed (n = 136, 10.7%), both parents unemployed (n = 125, 9.8%) and either of the parents in formal employment were the second highest grouping (n = 292, 23.0%). [Table 1].

**Table 1:** Sociodemographic profile of the children

Characteristics		Number	%
*Gender*			
	Male	634	49.7
	Female	641	50.3
**School**			
	A	291	22.8
	B	364	28.5
	C	188	14.7
	D	432	33.9
**Age** (*M* = 8.44, *SD* = 4.94)			
**Age Groups**			
	3– 5 years	159	12.5
	6– 9 years	737	57.8
	10– 12years	254	19.9
	≥ 13 years	125	9.8
**Level of education**			
	Grade R*	256	20.1
	Grade 1	287	22.4
	Grade 2	206	17.6
	Grade 3	124	9.1
	Grade 4	124	9.1
	Grade 5	97	7.6
	Grade 6	88	6.8
	Grade 7	93	7.3
**Living arrangement**			
	Both parents	358	28.1
	Either mother or father	133	10.4
	Other relatives	194	15.2
	Unspecified	590	46.3
**Parents/caregivers’ employment status**			
	Both parents employed	136	10.7
	Both parents unemployed	125	9.8
	Either mother or father employed	292	23
	Other sources of income including grants	722	56.5

* In South Africa, “Grade R” refers to the grade level that is equivalent to the reception year or the first year of formal schooling in the country’s education system. Grade R is the year before learners in South Africa start formal schooling and has been part of the General Education Training Band (GET) since 1998. It is also commonly known as “Grade RR” or “Reception Year.“ Grade R is part of the Foundation Phase, which is the first phase of the South African schooling system, catering to children from the age of 5 to 9 years old. During this phase, the focus is on developing foundational skills and preparing children for more formal learning in the later grades

### Nutritional characteristics of the children

We report results on nutrition: over and under-nutrition among the children. Out of the 1275 children’ screened, 55% of them weighed normal, 18% were at risk of being ‘overweight’, and 7% were ‘overweight’. ‘Severe stunting’ was found among 2%, 3% were underweight, whilst 3% were wasted [Table 2]. Relatedly, the ‘weight’ of the children ranged from 13 to 58 kg with an average weight of 27.15 kg (*SD* = 10.71). With regard to height, the children had a height range of 89 cm to168cm, with an average height of 123.83 cm (*SD =* 15.31) [Table 2].

**Table 2:** Nutritional status of school-going children

Variable		Number of Participants	%
Nutrition			
	Normal weight	767	55.0
**Over nutrition**			
	At risk of overweight	210	18.0
	Overweight	91	7.0
	Obese	18	2.0
**Undernutrition**			
	Underweight	37	3.0
	Stunting	127	10.0
	Severe stunting	29	2.0
	Wasting	37	3.0

### Demographic characteristics and nutritional status of school-going children

Pearson’s chi-square (χ^2^) test was conducted to examine the relationship between socio-demographics characteristics and nutritional status of school children.

### Children’s school and nutritional characteristics

**Fig. 1:**
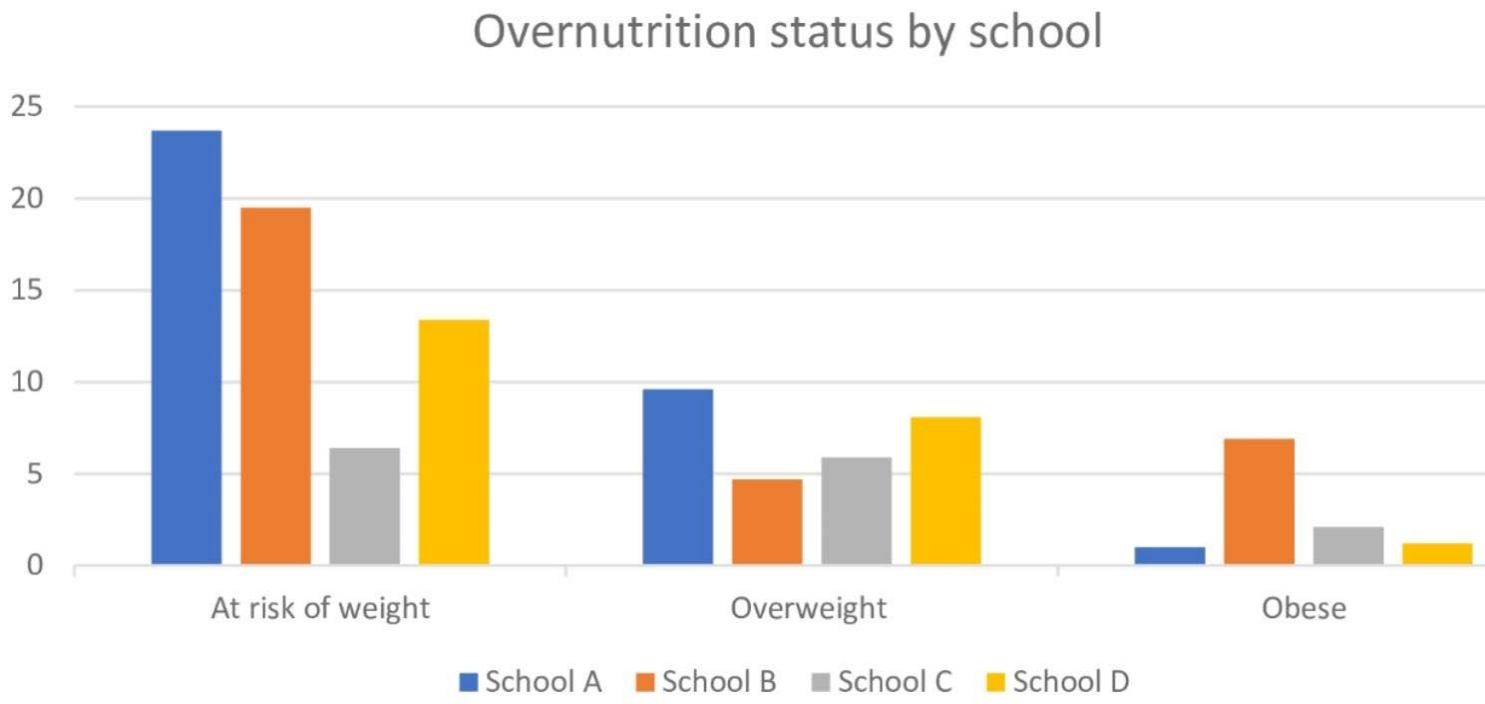
School-specific patterns of over-nutrition of school-going children

**Fig. 2:**
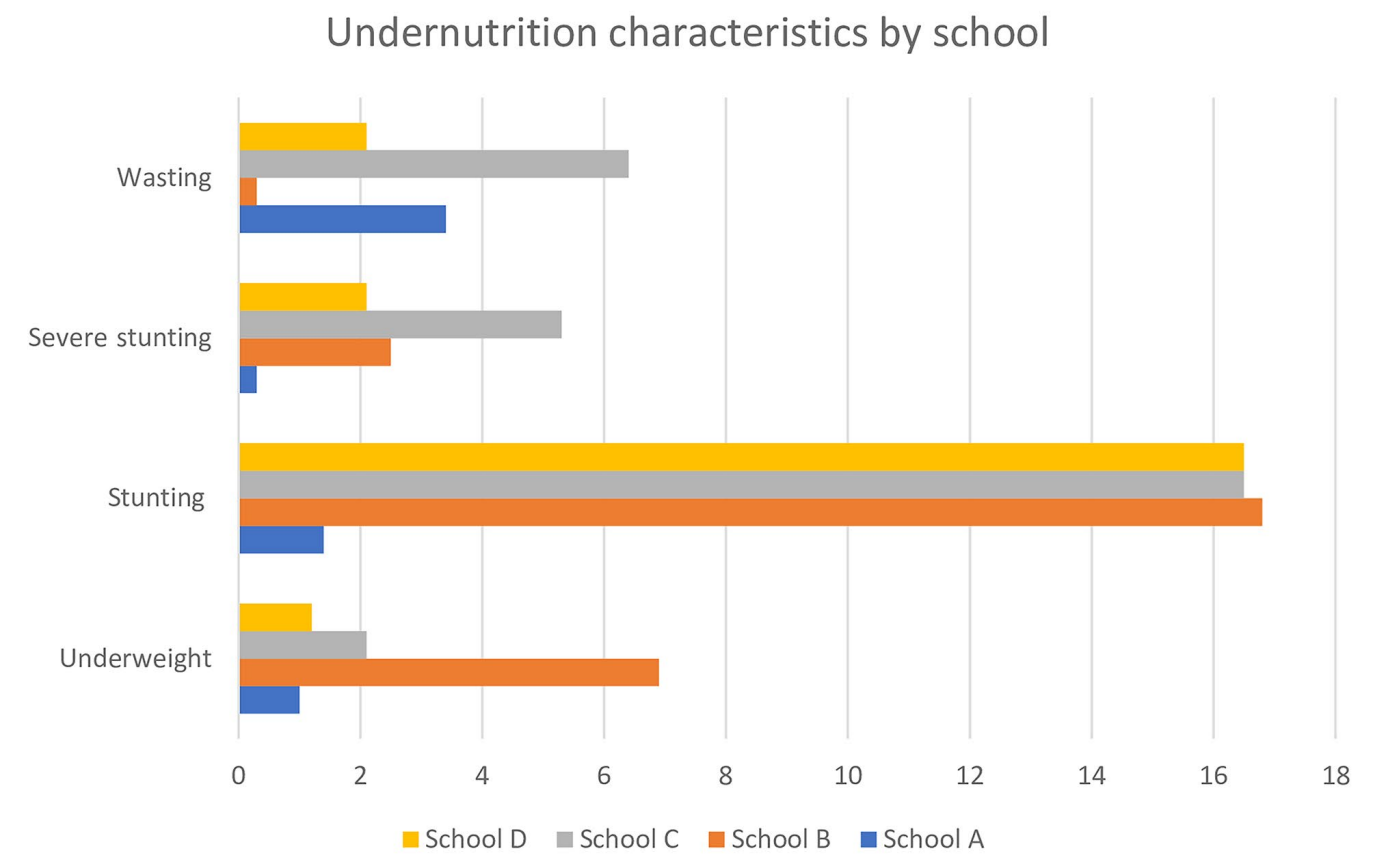
School-specific patterns of undernutrition of school-going children

The schools that the children attended had some statistically significant association with underweight (p = 0.000), at risk of overweight (*p* = 0.000), stunting (*p* = 0.000), severe stunting (*p* = 0.005), wasting (*p* = 0.010), and obese (p = 0.037). However, children from ‘School B’ primary school were significantly more likely to suffer from overweight, underweight, stunting, severe stunting and obesity compared to ’Schools A’, ‘School C’ and ‘School D’ [Figs. 1 and 2].

### Age, children’s grade, stunting and severe stunting

We also found an age-group difference in relation to children’s nutritional status. Age was significantly associated with both stunting (*p* = 0.041) and severe stunting (*p* = 0.012). Prevalence of stunting increased among school-going children within the ages 3–5 (8.2%) to 6–9 (8.6%) and further to 13.7% among children within the ages of 10-12years [Table 3]. The children’s level of education was statistically associated with stunting (*p* = 0.037). The same pattern was observed for children who suffered from severe stunting. Highest prevalence of stunting (20.5%) was recorded among children in Grade 6 compared to children in other grades [Table 3].

**Table 3:** Sociodemographic characteristics of school-going children and stunting, severe stunting, wasting and underweight

Characteristics		Stunting			Severe Stunting			Wasting			Underweight			
		Yes (%)	No (%)	(*p-*value)	Yes (%)	No (%)	(*p-*value)	Yes (%)	No (%)	(*p-*value)	Yes (%)	No (%)	(*p-*value)	
**Age Ranges**				**0.041**			**0.012**			0.795			0.071	
	3–5	8.2	91.8		1.3	98.7		3.1	96.9		1.9	98.1		
	6–9	8.4	91.6		1.9	98.1		3.1	96.9		3.9	96.1		
	10–12	13.7	86.3		2.0	98.0		2.0	98.0		1.6	98.4		
	≥ 13	13.6	86.4		6.4	93.6		3.2	96.8		0.8	99.2		
**Level of education (Grade)**				**0.037**			0.161			0.077			**0.018**	
	R	10.5	89.5		2.3	97.7		2.7	97.3		3.5	96.5		
	1	9.1	90.9		3.1	96.9		4.5	95.5		5.9	94.1		
	2	7.3	92.7		0.0	100.0		0.0	100.0		1.5	98.5		
	3	8.9	91.1		2.4	97.6		4.8	95.2		3.2	96.8		
	4	12.1	87.9		0.8	99.2		3.2	96.8		1.6	98.4		
	5	9.3	90.7		4.1	95.9		1.0	99.0		0.0	100.0		
	6	20.5	79.5		4.5	95.5		4.5	95.5		1.1	98.9		
	7	6.5	93.5		2.2	97.8		2.2	97.8		1.1	98.9		
**Living arrangements**				0.129			0.357			0.538			**0.028**	
	Both Parents	7.5	92.5		2.8	97.2		2.8	97.2		1.4	98.6		
	Either mother or Father	8.3	91.7		1.5	98.5		4.5	95.5		2.3	97.7		
	Other Relatives	13.4	86.6		3.6	96.4		3.6	96.4		1.5	98.5		
	Unspecified	10.7	89.3		1.7	98.3		0.2	97.6		4.4	95.6		
**Parents/caregivers’ employment status**				0.100			0.178			**0.019**			**0.033**	
	Both Parents employed	6.6	93.4		0.0	100.0		1.5	98.5		0.0	100.0		
	Both parents unemployed	14.4	85.6		2.4	97.6		7.2	92.8		1.6	98.4		
	Either mother or father employed	7.9	92.1		3.4	96.6		3.1	96.9		2.1	97.9		
	Social grant	50.7	49.3		0.3	99.7		0.1	99.9		4.0	96.0		

### Children’s living arrangement, their weight and parents’ employment

School-going children’s living arrangements had a statistically significant association with normal weight *(p* = 0.000), at risk of underweight (*p* = 0.000), and underweight (*p* = 0.028). It was observed that the prevalence of normal weight decreased from 62.0% for school children aged 6–9 years to 58.6% for those aged 10–12 years and further to 52.0% for children aged 13 years and above [Table 4]. Overweight ranged from 6.4 to 8.8% by age and was highest in Grade 4 at 10.5%. Children who did not specify their living arrangement were more likely to be at risk of being overweight and underweight compared to the children that lived with both parents (22.2% vs. 12.3%) and (4.4% vs. 1.4%), respectively [Table 4].

Besides, parents or caregivers’ ’employment status’ was also significantly associated with normal weight (*p* = 0.000), at risk of underweight (*p* = 0.001), underweight (*p* = 0.033) and obesity (*p* = 0.019). Children who had both parents employed were more likely to have normal weight (75.0%) compared to those who depended on social grant (55.4%). A further analysis also revealed children whose parents/caregivers were unemployed suffered wasting (7.2%) compared to children who had both parents employed (1.5%). However, the social grant to parents/caregivers showed some positive effects on the number of children who had normal weight (55.4%), wasting (1.0%), underweight (4.0%), ‘at risk of overweight (20.2%)’ [Table 3].

**Table 4:** Sociodemographic characteristics of school-going children and their weight characteristics

Characteristics		Normal weight			At risk of overweight			Overweight		
		Yes (%)	No (%)	(*p-*value)	Yes (%)	No (%)	(*p-*value)	Yes (%)	No (%)	(*p-*value)
**Age Ranges**				0.156			0.122			0.633
	3–5	59.1	40.9		20.8	79.2		8.8	91.2	
	6–9	62.3	37.7		15.1	84.9		6.4	93.6	
	10–12	58.6	41.4		15.2	84.8		8.2	91.8	
	≥ 13	52.0	48.0		21.6	78.4		7.2	92.8	
**Level of education (Grade)**				**0.017**			0.129			0.278
	R	57.0	43.0		18.8	81.3		9.4	90.6	
	1	60.3	39.7		13.6	86.4		4.5	95.5	
	2	68.0	32.0		16.5	83.5		6.3	93.7	
	3	64.5	35.5		10.5	89.5		5.6	94.4	
	4	58.1	41.9		15.3	84.7		10.5	89.5	
	5	59.8	40.2		18.6	81.4		6.2	93.8	
	6	44.3	55.7		25.0	75.0		6.8	93.2	
	7	63.4	36.6		18.3	81.7		9.7	90.3	
**Living arrangement**				**0.000**			**0.000**			0.747
	Both parents	66.5	33.5		12.3	87.7		7.8	92.2	
	Either mother or father	66.9	33.1		12.0	88.0		6.0	94.0	
	Other eelatives	65.5	34.5		9.8	90.2		5.7	94.3	
	Unspecified	53.1	46.9		22.2	77.8		7.5	92.5	
**Parents/caregivers’ employment status**				**0.000**			**0.001**			0.198
	Both parents employed	75.0	25.0		11.0	89.0		5.9	94.1	
	Both parents unemployed	62.4	37.6		11.2	88.8		3.2	96.8	
	Either mother or father employed	64.0	36.0		12.0	88.0		8.9	91.1	
	Social grant	55.4	44.6		20.2	79.8		7.3	92.7	

## Discussion

The aim of this retrospective descriptive cross-sectional study was to ascertain the nutritional status of school-aged children in the Marianhill area, in South Africa. We assessed the association between sociodemographic characteristics of school-going children and their nutritional status. We report the findings under three themes: school-going children’s level factors, school level factors and parents/caregivers level factors relative to nutritional status of the children.

### School-going children’s level factors

Nutrition among school-going children in LMICs is an important issue that affects both their physical and mental development. According to the WHO classification for BMI-for-age, 55% of children, in our study, were of normal weight, while the prevalence of overweight and obesity ( > + 2 SD) was relatively low (7%). However, the percentage of children considered to be at risk for being overweight was significantly high (18%) relative to global proportion of children aged 2–19 years in 2020 [27]. The coexistence of undernutrition among these children, along with the number of wasted (<-2 SD) and severely wasted (<-3 SD) children (5%) confirms the double burden of over- and undernutrition prevalent in South Africa and other LMICS [10, 11]. The majority of the participants in this study were within the of 6 to 9 years, with a mean age of 8.4 years. The age of the children had a statistically significant relationship with stunting (p = 0.041) and severe stunting (p = 0.012), but not with wasting, normal weight, ‘at risk of being overweight,‘ or underweight. School-going children within the ages of 10 to 12 were significantly more stunted than those of other age groups. Malnutrition was prevalent among all age groups of school-aged children. These findings highlight the fact that, despite relatively low levels of wasting and severe wasting, the observed at ‘risk for obesity’ may suggest the prevalence of persistent nutritional issues among school-aged children in this study. Armstrong, Lambert and Lamber [28] reported moderate stunting among 7–10-year-olds, in a similar study conducted in South Africa.

Literature suggests that chronic malnutrition and stunting are prevalent during infancy among children living in environments with limited resources [29]. However, previous anthropometric research in QwaQwa, South Africa, revealed that 11.3% of the population was stunted and 2.8% were severely stunted [30]. Studies show that a significant proportion of school-age children in LMICs were stunted, with lower academic performance and more likely to miss school due to illness [31–33]. Overall, stunting remains a chronic public health issue in the majority of low- and middle-income countries, and given that these children attend school, there is a need for immediate policy intervention to address the problem.

### School-level factors

The bivariate analysis of this study revealed a statistically significant association (p < 0.005) between the children’s schools and underweight, stunting, severe stunting, and wasting. Children at ‘School B’ primary were significantly more likely to be underweight, stunted, or severely stunted than children at the other three institutions. These statistics may suggest that ‘School B’ primary school children are from more deprived communities than children at other schools. Geographical and school-level disparities in school-going children’s nutritional problems related to social and economic issues, such as a high poverty rate, unemployment, abuse, HIV/AIDS, have been identified in prior researches [34]. These disparities may negatively impact communities and parents or guardians’ ability to meet the nutritional needs of their school-going children [34]. In a systematic review in South Africa, Mkhize and Sibanda [9] reported that Limpopo Province, Gauteng Province (11.88%), Eastern Cape Province (14.46%), and KwaZulu-Natal Province (15.25%) had the highest prevalence of underweight children. The prevalence of overweight or obesity was high in the Western Cape (10.23%), and it was also high in the Northern Cape (22.20%). These findings at the school level underscores the even more of extreme poverty among the deprived and poor in the study schools. Some studies have revealed that beneficiaries of the programme are sometimes malnourished, given poor-quality food, or go without sustenance for some days [35, 36].

There is a need for organisations that are supporting schools with nutritional needs, as well as the government and other stakeholders in the Integrated School Health promotion programme to establish appropriate and adequate policies to guide the implementation, monitoring, and evaluation of the school feeding programme. Providing nutritious meals in schools necessitates an ongoing evaluation to ensure nutritional sufficiency. Several nations offer school nutrition programmes for children older than 5 years. As a means of enhancing the dietary diversity of pupils, the school feeding programme in southern Ethiopia introduced new food groups into their diet. This resulted in enhanced nutritional status, increased school attendance, and decreased dropout rates among beneficiaries of school feeding programmes [37].

### Parent/caregiver level factors

‘Living arrangements’ of the children was significantly associated with ‘normal weight,‘ ‘at risk of underweight,‘ and ‘underweight’ among children. Even children whose living arrangement was not specified during the screening exhibited “risks of being overweight” and “risks of being underweight.“ The employment parents or caregivers were engaged in, was found to be significantly associated with normal weight, risk of underweight, underweight, and obesity (p = 0.019) [Tables 3 and 4]. As a result, children whose parents or primary caregivers held formal employment were more likely to be of normal weight (75%) than those who relied on social grants (55.4%). Indeed, the highest risk of overweight was in those who received a social grant at 20.2% in this study. Families receiving social grants may have low-paying jobs or no formal employment, limiting access to healthier food options and recreational facilities. Processed, calorie-dense foods may be more affordable, leading to unhealthy diets [26].

Children whose parents or guardians were not formally employed were positively associated with ‘wasting’. Consistent with a previous study, children in 26 public schools in Bloemfontein whose parents held skilled and graduate-level jobs had a higher prevalence of stunting. Significantly more children of graduate parents (8.1%) were overweight or obese than those of less-educated parents (6.9%) [26]. Overall, the employment status of a child’s parents was not associated with wasting, obesity, stunting, or severe stunting in that study [26].

Previous studies in South Africa found relatively insignificant differences in nutritional status between orphans and non-orphans [7]. Poor health status has been found to be associated with lower quality of life, decreased utilisation of health services by adolescents, and increased morbidity and mortality [7, 38]. Overall, our findings and those of previous research works on school-going children’s nutritional status suggest that the problem of malnutrition continues to affect school-going children in South Africa, with potential consequences for learning outcomes.

Incorporating nutrition into primary healthcare has improved child nutrition in Bangladesh, Thailand, with comparable outcomes in other LMICs [39–41]. The early implementation of nutrition programmes can reduce the risk of stunting by a greater amount and at a lower cost. Positive outcomes for early childhood development have been linked to early provision of optimal nutrition and learning opportunities supported by responsive carer practices [39–42]. Consequently, school-going children’s awareness of the foods they consume outside, and the dietary implications may need to be incorporated into the health education curriculum in order to reduce their exposure to harmful foods.

The South African government deserves commendation for developing a number of interventions to combat household food insecurity. Consequently, the government established the school nutrition programmes, child support grants, and old-age pensions. However, food variety and dietary diversity, which are related to the nutritional status of South African children, are limited in poor communities [10, 11]. The quality and variety of food available to children living in poor communities may also be a factor in this sample’s undernutrition. According to the available evidence, food insecurity may be associated with both types of malnutrition [4, 6]. On this basis, selective intervention programmes, such as food garden projects and other community projects, are required; selective intervention programmes target children who are most at risk of underweight, stunting, and wasting.

### Implications for policy and practice

#### Parents and neighbourhoods

Proper nutrition is an essential intervention. Based on the findings, there is a need for NGO and CBO-facilitated community empowerment programmes in the respective communities. These organisations could conduct nutrition workshops in the community in conjunction with nutritionist and public health nurses at local clinics. In addition, nutrition and education information helps mothers and other family members understand how to keep their children healthy, why their children may not be growing properly, and how to treat them. This study’s findings could aid in the revision of current public health policy to improve the nutritional status of children in South African schools. In addition, the findings of this study could provide useful surveillance data for public health action/intervention for this age group and inform the development of health promotion and education strategies for school-aged children.

#### School health

Based on the findings, we urge community health sector stakeholders to implement interventions that will increase the number of healthcare professionals within school health teams. The findings also indicate that there is an urgent need for community health workers to receive training in order to conduct home visits to families with children who have nutritional problems. Community health workers could educate families on the importance of nutrition and maintaining a balanced diet during home visits.

### Limitations of the study

The limitations of the study must be considered when interpreting its results. First, the findings were based on children who presented for screening in these four schools during the specified time frame. Consequently, there might have been some children with worse nutritional conditions or an improved nutritional status who did not participate in the screening interventions, which could influence the strength of the associations between the variables. The findings were also based on selected schools that benefited from a community-based organization’s intervention programme. Thus, these results may differ from those of children in other schools in the same geographic region who did not receive the intervention. We observed that school nurses did not provide information on the living situations of all children, which could have an impact on the findings. In order to avoid erroneous conclusions in this regard, we opted to use only the most basic associations between the variables. The study also intend to disseminate the findings to the schools. Therefore, during the dissemination workshop, nurses will be informed of the importance of accurately completing all sections of the form during interventions. Thirdly, sociodemographic and economic indices and nutritional characteristics of children were not segregated by race. This is because the purpose of the study was to highlight the nutritional status of children, and therefore no racial data was collected. Nevertheless, given the consistency of the findings with previous research in South Africa, it is highly probable that the findings reflect the current nutritional situation of children in the study communities.

## Conclusion

This study provides evidence of malnutrition (under-nutrition and over nutrition) among school-going children in the Marianhill area, Durban Metropolis. The findings revealed an unacceptable prevalence of stunting, and overweight, exist among the children. The findings highlight the importance of school screening of school-going children for nutritional health status as it plays an important role in determining children’s health, physical growth and development, academic performance, and progress in life. The screening provides a window of opportunity to address nutritional problems in school-going children early on. Indeed, whether steps were taken to address these nutritional problems among school-going children in the study communities requires further investigation. We also recommend evaluation into the impact of the social grants on school-going children’s nutritional characteristics within the study area. We recommend policy reforms to address the nutritional status of school-going children, particularly, because of the potential economic impact of the social grants on nutritional status of children in this study. We also recommend a qualitative study into the lived experiences of parents and caregivers concerning nutritional issues among children at the household level.

### Electronic supplementary material

Below is the link to the electronic supplementary material.

Supplementary Material 1

## Data Availability

Data is readily available upon reasonable request from the corresponding author.

## Data Availability

The datasets used and/or analysed during the current study is available from the corresponding author on reasonable request.

## Abbreviations

**CBO ** Community: Based Organisation
**ISHP**: Integrated School Health Programme KZN KwaZulu-Natal
**LMICs**: Low and Middle Income Countries
**NGO**: Non-Governmental Organisation
**NSNP**: National School Nutrition Programme
**SADHS**: South African Demographic and Health Survey
**SANHANES**: South African National Health and Nutrition Examination Survey
**WHO**: World Health Organization
