# Supplementary figures and images for: Factors associated with underweight, overweight, stunting and wasting among primary school-going children participating in a school health initiative in South Africa
Source: BMC Nutr. 2023 Oct 25;9:119. doi: 10.1186/s40795-023-00778-x (PMC10601266; doi:10.1186/s40795-023-00778-x)

Supplemental file: School-going children’s (Leaner) Assessment Form


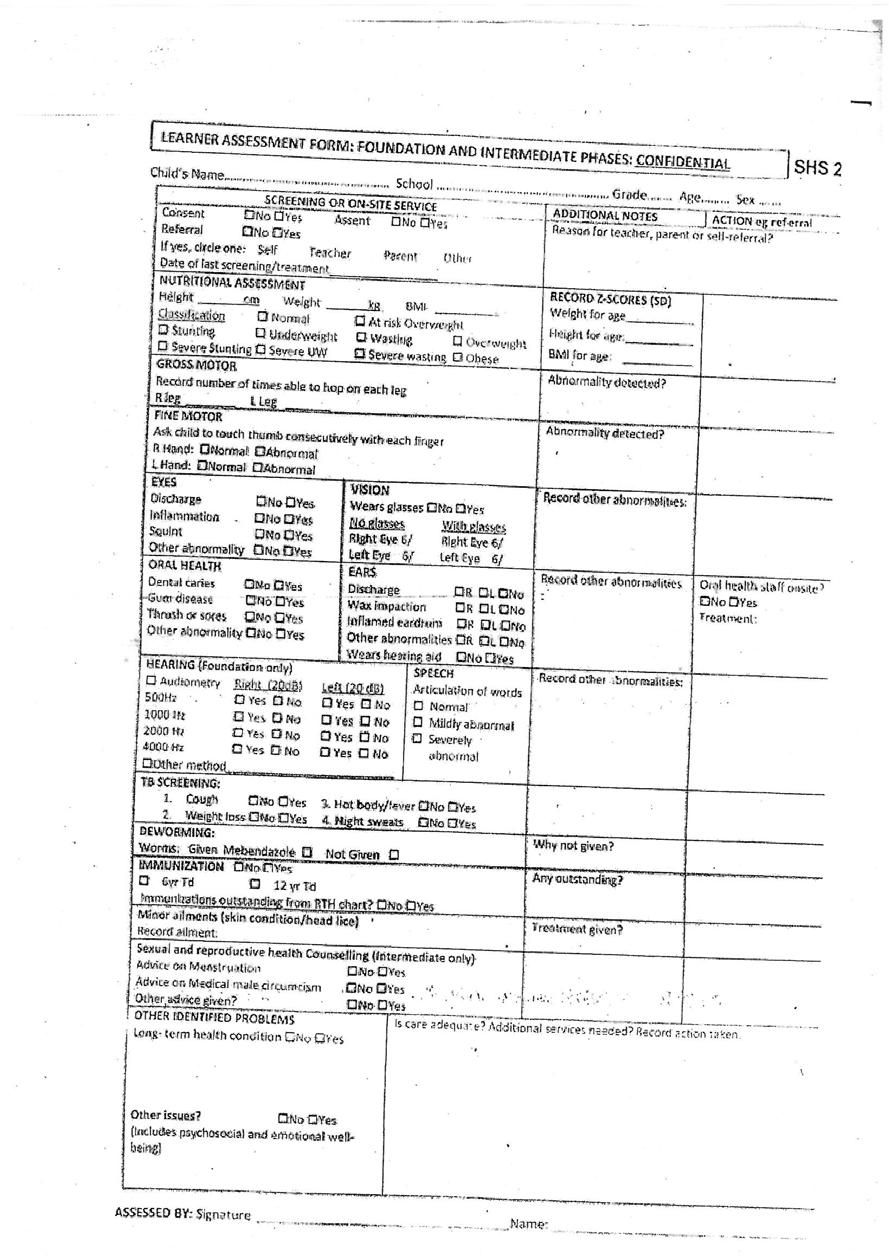

Supplement: Supplementary file 1 — Supplementary Material 1 [file 40795_2023_778_MOESM1_ESM.docx]
